# Exploring the nature of music-evoked autobiographical memories in healthy aging: A mixed-methods study

**DOI:** 10.1177/10298649241297931

**Published:** 2025-08-25

**Authors:** Mia O’Shea, Ilja Salakka, Anni Pitkäniemi, Emmi Pentikäinen, Petri Toiviainen, Teppo Särkämö

**Affiliations:** University of Helsinki, Finland; University of Helsinki, Finland; University of Helsinki, Finland; University of Helsinki, Finland; University of Jyväskylä, Finland; University of Helsinki, Finland

**Keywords:** autobiographical memory, content analysis, listening, reminiscence bump, music-evoked emotions

## Abstract

Autobiographical memories play a significant role in shaping a sense of self and are essential for normal functioning on a daily basis. Music-evoked autobiographical memories (MEAMs) are memories of past events that are triggered by music, often involuntarily. In this study, we had two aims: (1) to explore the qualitative characteristics of MEAMs in healthy aging and (2) to analyze possible relationships between types of MEAM and the emotions evoked by music. We played 140 excerpts from songs from the four decades between 1950 and 1990, and folk songs, to 82 healthy adults aged 60–86 years and collected their MEAMs, expressed in their own words. We applied content analysis to the data and categorized them manually. Participants rated the valence, arousal, and intensity of emotions evoked by each excerpt. We conducted a mixed-effects logistic regression analysis to identify relationships between participants’ MEAMs and ratings. Participants frequently reported music-related activities such as singing, dancing, and listening, and specific people and places, in their MEAMs. The excerpts evoked more autobiographical memories from participants’ youth than from any other periods in their lives. Our analysis of the relationships between types of MEAM and the emotions evoked by music suggest a variety of emotional profiles. The choice of methods emphasized ecological validity and the findings provide a new perspective on the nature of MEAMs in healthy aging.

Hearing a certain piece of music can instantly transport an idle mind back to a particular episode from the past, reminding the hearer of long-forgotten details associated with that particular musical memory. These types of memories are known as music-evoked autobiographical memories (MEAMs), defined as recalled autobiographical memories of past events that are triggered by musical stimuli, often involuntarily (Cuddy et al., 2017; Rasmussen & Berntsen, 2009). At the neural level, the process whereby autobiographical memories are evoked is thought to occur because of the complex way in which music simultaneously activates multiple interacting brain networks associated with memory, emotion, perception, sensory, and motor functions (Janata, 2009). Overall, music has been found to be an effective cue for retrieving autobiographical memories (Cady et al., 2008; Scherer & Zentner, 2001).

Nevertheless, it is important to consider what is special about music and that might make it more effective than other cues. There is some limited evidence from comparisons of studies in which autobiographical memories were evoked by musical cues (Schulkind & Woldorf, 2005) and word cues, respectively (Rubin & Schulkind, 1997), such that a greater proportion of autobiographical memories were evoked by the musical cues than the word cues. This may have been because there were methodological differences between the two studies and also differences between them in terms of the emotionality of the cues (Schulkind & Woldorf, 2005). All the same, a greater proportion of autobiographical memories were evoked by musical cues than food cues (Jakubowski, Belfi, et al., 2023). Yet musical cues may not be more effective than visual, word, and sound cues (Belfi et al., 2016; Jakubowski & Eerola, 2022; Jakubowski, Walker, & Wang, 2023) when the outcome measure is total number of memories recalled, or the ease with which they are recalled. However, music-evoked memories tend to be more emotional than memories evoked by other types of cue. This is seen especially in the valence of cue-evoked memories: those evoked by music are more pleasant than those evoked by other types of cue (Jakubowski & Eerola, 2022; Janata et al., 2007; Jakubowski, Walker, & Wang, 2023). While Mehl et al. (2024) found that music that evokes memories is more likely to elicit negative emotions than music that does not evoke memories, they also found that music evokes more positive than negative emotions overall. Music-evoked memories are not only reported to be strongly emotional but also vivid (Belfi et al., 2016; Janata et al., 2007).

The relationship between MEAMs and music-evoked emotions is likely to be two-way. On the one hand, MEAMs can evoke emotions, but emotions may help the consolidation and retrieval of memories (Kensinger, 2009; Schulkind et al., 1999). On the other hand, music can evoke memories and emotions independently of each other. Thus, if a person were asked about the emotions evoked by a particular piece of music, they would be making a valid response if they reported not only the emotions evoked by the music but also the emotions evoked by the MEAMs. If the person were asked about the emotions evoked by a particular MEAM, those emotions would have to be separated from the emotions evoked by the music, rather than the MEAM and music in combination.

There are strong connections between the music that an individual has listened to during key episodes in the individual’s life and their sense of self and autobiographical memory (Batcho, 2007). Sense of self is composed of memories of events and experiences over the course of life, which contributes to the formation of a cohesive personal identity (Conway, 2005). Autobiographical memory is a distinct type of self-referential memory that expands a network of personal experiences and episodes collected throughout an individual’s life as it is experienced subjectively (Vanderveren et al., 2017). Autobiographical memories can contain various types of detail from episodic memory, such as specific life experiences, people, events, locations, or objects, as well as details from semantic memory, such as general knowledge about the world or information about oneself (Williams et al., 2008). In what is known as the *reminiscence bump*, people usually find it easiest to retrieve autobiographical memories from their youth, a phenomenon that has been repeatedly observed in the context of music (Jakubowski et al., 2020; Krumhansl & Zupnick, 2013; Platz et al., 2015; Schulkind et al., 1999; Schulkind & Woldorf, 2005).

Autobiographical memory is closely associated with emotions. This association is particularly evident in the case of MEAMs, which are intimately linked to the emotions evoked by the songs. Emotions, including those evoked by music, can be viewed on a continuum in terms of their valence, from unpleasant to pleasant; on a continuum in terms of their intensity, from weak to strong; and producing arousal on a continuum from low to high (Posner et al., 2005; Russell, 1980; Russell & Carroll, 1999). Neuroimaging studies have shown that familiar music activates the limbic/paralimbic and medial frontal regions and reward circuitry in the brain, all related to emotions (Altenmüller et al., 2014; Janata, 2009; Pereira et al., 2011), suggesting a close neural coupling of emotions and autobiographical memory when the individual listens to music. In our previous study with healthy older adults (Salakka et al., 2021), we observed very strong associations between the emotional intensity and valence of a song, and the arousal it induces, and its autobiographical salience. Autobiographical memory is essential for several different cognitive functions supporting overall well-being and the effective functioning of the self (Vanderveren et al., 2017). For example, autobiographical memory provides essential information about past events that individuals can compare with present events. It can thus help to direct individuals’ current behavior or predict their future behavior (Baddeley, 1987). Autobiographical knowledge is also essential for building a consistent life timeline and sense of self so that individuals can realize who they were before, who they are now, and who they could be in the future (Conway, 2005). Autobiographical memories additionally serve to create and strengthen social bonds between people by facilitating social interaction, fueling conversations, and enabling the sharing of experiences as well as increasing intimacy and sympathy toward others (Williams et al., 2008). In this study, we define *autobiographical memory* as any kind of self-referential information that is related to the past.

As people get older and make the transition from employment into retirement, they increasingly report the use of music specifically for emotional self-regulation (Saarikallio, 2011). Older adults also report being motivated to listen to music from day to day because it can evoke autobiographic memories (Laukka, 2007). They often consider music to be a meaningful component of daily life, and therefore use it for relaxation, revival, entertainment, and even to alleviate feelings of loneliness (Saarikallio, 2011). MEAMs in healthy older adults have been reported to be more positive, in general, than those of younger adults (Ford et al., 2016; Jakubowski et al., 2021; Jakubowski, Belfi et al., 2023). While gradual neurodegeneration occurring with aging can impair many cognitive functions, MEAMs have been found to be largely spared from deterioration in older adults (Collett et al., 2012) and have also been observed in persons with severe acquired brain injury (Baird & Samson, 2014) and Alzheimer’s disease (AD; Cuddy et al., 2015; El Haj et al., 2012; Kaiser & Berntsen, 2023). Studies in which healthy older adults were compared with people with AD in terms of MEAM content have found mixed evidence regarding the emotionality of MEAMs. El Haj et al. (2012) found that both healthy older adults and people with AD produce more positive than negative words, on average, when describing their MEAMs, but people with AD produce more emotional words, both positive and negative, than healthy older adults. In another study, however, people with AD produced fewer positive words when describing their MEAMs than healthy older adults (El Haj et al., 2012). In both studies, autobiographical memories were more positive when they were elicited after a period in which music was played than when they were evoked after a period of silence. It may be that singing and listening to familiar songs helps to preserve autobiographical memory in people with dementia (Särkämö et al., 2014). Given that MEAMs appear to remain intact in diverse aging populations, it is worth incorporating activities involving music in rehabilitation and care to support well-being until the very late stages of life.

The emergent need, worldwide, to make use of cost-effective, non-invasive, and non-pharmacological treatments for the rehabilitation and care of older adults, and the potential for regular musical activities to support memory in aging (Gooding et al., 2014; Mansens et al., 2018) and dementia (Lyu et al., 2018; Pongan et al., 2017; Särkämö et al., 2014), have highlighted the importance of further investigating the nature of MEAMs and finding ways in which music evoking autobiographical memories could be used in healthcare to improve the daily lives of older adults. In this study we aimed to advance the general understanding of MEAMs by (1) exploring the qualitative characteristics of MEAMs in healthy aging and (2) analyzing possible relationships between types of MEAM and the emotions evoked by music. We addressed the first aim by asking about the kinds of information and levels of detail contained in the MEAMs of healthy older adults, whom we defined for the purposes of the present study as individuals aged 60 years and above. We also predicted that there would be a greater density of reported memories from the period of youth, that is, a reminiscence bump. We addressed the second aim by conducting an exploratory mixed-effects logistic regression analysis to identify relationships between the content of participants’ MEAMs and ratings of the emotions evoked by the excerpts from songs they had heard.

## Methods

### Participants

A total of 113 participants carried out a task in which they rated excerpts from songs that were classified as old-time music (*old-time music-rating task, or* OMRT), and a subset of 82 participants also provided verbal descriptions of the memories evoked by the excerpts (*memory inserts*; see Table 1 for demographic information).

**Table 1.** Age, gender, and choir-singing background for all participants and those who submitted memory inserts.

	All participants who did the OMRT (*N* = 113)	Participants who submitted memory inserts (*n* = 82)
Age	*M* = 70.76, *SD* = 5.38, range = 60–86	*M* = 70.51, *SD* = 4.91, range = 60–85
Gender	Women: *n* = 86 (76.1%), Men: *n* = 27 (23.9%)	Women: *n* = 65 (79.3 %), Men: *n* = 17 (20.7%)
Choir-singing background	Choir singers: *n* = 78 (69.0%)	Choir singers: *n* = 57 (69.5%)

Participants were all native Finnish speakers and none of them had a history of neurological, psychiatric, or substance abuse disorders at the time of the study. They were recruited through Helsinki, Vantaa, and Espoo Adult Education Centers as a part of a larger cohort study aiming to determine the long-term benefits of singing in a senior choir on cognitive, emotional, and social functioning (Pentikäinen et al., 2021, 2022, 2023). The study was approved by the Ethical Review Board in the Humanities and Social and Behavioural Sciences of the University of Helsinki, and all participants gave written informed consent.

### Data collection

The data were collected through the OMRT, which was implemented as a web browser application (*app*) designed for this project in collaboration with Sentina, a Finnish company. Participants could complete the OMRT whenever and wherever convenient to them, using their personal computer or a tablet computer provided by the researchers. The task was conducted entirely in Finnish. Before starting the actual task, participants did a short test trial to get accustomed to the user interface, set their output and input volumes to suitable levels, and practice submitting an autobiographical memory in writing or using the microphone to make an audio recording.

### Procedure

The OMRT presented participants with excerpts from 140 songs classified as old-time music, in genres including traditional folk, popular, rock, and jazz. The excerpts were 30 s long, on average (range = 18–37 s). The songs had been selected from a list of 225 songs taken from the archives of main Finnish radio channels. Following a pilot study, songs with very high familiarity and autobiographical salience were excluded. The final selection of songs was divided into two lists (A and B) each containing 15 songs that were popular in each of the four decades from 1950 to 1990 (50s, 60s, 70s, 80s) plus 10 folk songs. The 70 excerpts in each list were matched for genre, familiarity, and autobiographical salience, based on the results of the pilot study (see Supplemental Material 1 for more information). Participants were assigned to List A or B, matched for age, gender, and choir-singing background.

After hearing each excerpt, participants were asked to rate it using 5-point Likert-type scales measuring five dimensions: valence, emotional intensity, arousal, familiarity, and autobiographical memories (see Supplemental Material 2 for the questions and anchors for each scale, and Salakka et al., 2021 for the results of this part of the larger study). The questions were presented sequentially after each excerpt in the same order. The app allowed participants to move to the next excerpt when they had answered all five questions, although they could play excerpts again while they were answering the questions.

In the present study, we used the ratings of valence, emotional intensity, and arousal (*emotion ratings*) to analyze their relationships with the contents of participants’ MEAMS. We did this because we wanted to explore the emotions evoked in participants by the music rather than the emotions evoked by their autobiographical memories, which might or might not have been independent of each other. We also offered participants the opportunity to provide memory inserts. The instruction for this was simply “Submit a memory,” and the participant either typed it into a text box or made an audio recording in the app.

### Data analysis

As well as using the emotion ratings of the excerpts in a mixed-effects logistic regression analysis, we analyzed the content of the memory inserts. First, the first author (MO) categorized them, in part subjectively, as the categories emerged from the data, and in part to represent the three levels of autobiographical knowledge proposed by Conway and Pleydell-Pearce (2000): event-specific memories, general events, and lifetime periods. She found that they included not only descriptions of MEAMs, as intended (*MEAM inserts*), but unexpected content such as opinions, details of associations, and other semantic information (*non-MEAM inserts*). MO’s decisions were then reviewed by and discussed with the third author (AP) before being clustered, finally, into the overarching categories illustrated in Table 2. The sub-categories are not mutually exclusive, so the same memory insert could be assigned to more than one.

**Table 2.** Names and definitions of categories and sub-categories.

Category	Sub-category	Content of insert
Action memory	Dancing	A memory of dancing
	Singing	A memory of singing
	Performing	A memory of performing
	Listening	A memory of listening to the song
	Playing (instrument)	A memory of playing the song on an instrument
Specific detail	Location-specific	A specific location
	Object-specific	A specific object
	Person-specific	A specific person/people
	Event-specific	A specific event
	General event	A recurring event
Lifetime period		A distinguishable and themed time of life
	Childhood	Period of childhood
	Youth	Period of youth
	Adulthood	Period of adulthood
	Old age	Period of old age
Semantic content	Artist-related content	Information about or mention of artist/song composer
	General information	General knowledge, facts or claims
Associative detail	Association	A general association/mental connection
	Feeling	A memory of a feeling/emotional experience
	TV/Cinema/Radio	A memory of watching TV, going to the cinema or listening to radio
Other categories	MEAM insert	MEAM-related content
	Non-MEAM insert	Not MEAM related content
	Opinion	A personal opinion
	Multiple memories	Description or mention of more than one memory

Lifetime periods were sub-categorized as childhood, youth, adulthood, and old age, rather than in terms of particular years. Memory inserts were not assigned to a lifetime period unless the participant explicitly described a memory from one of these periods. Childhood memories included playing and the early years of school; memories of youth (Finnish: *nuoriso*, which typically excludes the pre-teenage years) included participants’ descriptions of being adolescents and students; memories from adulthood included working life, family life, parenthood, and being middle-aged; memories of old age included post-retirement and even recent events for older participants.

All statistical analyses were carried out using R version 4.2.2. For the purposes of analysis, where we wanted to compare the numbers of MEAM inserts associated with excerpts from songs that were popular in each of the four decades (50s, 60s, 70s, and 80s) and folk songs, we weighted the number of inserts referring to folk songs by a factor of 1.5, because there were fewer folk songs than songs from the 50s/60s/70s/80s in the original pool of 225 songs. The Kolmogorov-Smirnov (K-S) test from *stats*-package was used to compare the distribution of inserts to simulated distributions (R Core Team, 2022). Relationships between emotion ratings and MEAM-insert categories were assessed using a mixed-effects logistic regression utilizing *lme4* R-package (Bates et al., 2015) and using α = .005 as a conservative threshold due to the exploratory nature of these analyses. For drawing graphical plots, *ggplot2* R-package was used (Wickham, 2016).

## Results

### Categorization of inserts

**Figure 1.**
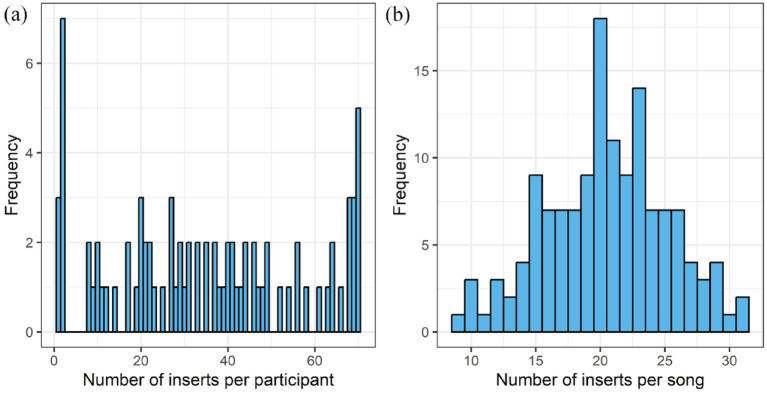
Distributions of memory inserts between participants and between song excerpts. (a) Distribution of inserts submitted by individual participants (*n* = 82), *M* = 34.96, *SD* = 22.04, K-S test against simulated uniform distribution (*n* = 10^5^) *D* = .11, *p* = .31. (b) Distribution of inserts submitted for individual excerpts (*n* = 140), *M* = 20.49, *SD* = 4.73, K-S test against simulated normal distribution (*n* = 10^5^) *D* = .08, *p* = .34.

Eighty-two of the 113 participants (72.57%) submitted one or more written or spoken inserts after hearing each of the 70 excerpts. As shown in Table 1, this subset of participants shared similar demographic characteristics to the original sample (reported in Salakka et al., 2021). They submitted a total of 2,782 inserts (range = 1–70) associated with 48.47% of the excerpts heard by each participant, on average. As shown in Figure 1(a) the distribution was relatively uniform, other than the small peaks at either end, showing that participants did not submit a typical number of inserts. Overall, 35.17% of the 70 excerpts heard by all 113 participants, a total of 7,910, prompted inserts, and 19.72% of 7,910 excerpts prompted MEAM inserts. The number of inserts associated with individual excerpts was relatively normally distributed with the most typical number being 20 (see Figure 1(b)). The mean number of words for a single insert was 17.42 (*SD* = 13.17, range = 1–137).

The numbers and percentages of MEAM and non-MEAM inserts (*N* = 2,782) assigned to the categories and sub-categories (Table 2) are shown in Table 3. Each insert was assigned to a mean number of 2.8 categories (range = 0–10, *SD* = 1.91). Non-MEAM inserts constituted 43.9% of all inserts and having been categorized as such were not analyzed further.

**Table 3.** Numbers and percentages of inserts assigned to each category and sub-category.

	MEAM inserts(total = 1,560)	Non-MEAM inserts(total = 1,222)	All inserts (total = 2,782)
Action memory	796 (51.0%)	34 (2.8%)	830 (29.8%)
Singing	379 (24.3%)	6 (0.5%)	385 (13.8%)
Listening	224 (14.4%)	27 (2.2%)	251 (9.0%)
Dancing	222 (14.2%)	1 (0.1%)	223 (8.0%)
Performing	37 (2.4%)	0 (0.0%)	37 (1.3%)
Playing (instrument)	24 (1.5%)	0 (0.0%)	24 (0.9%)
Specific detail	546 (35.0%)	14 (1.1%)	560 (20.1%)
Person-specific	336 (21.5%)	11 (0.9%)	347 (12.5%)
Location-specific	138 (14.2%)	1 (0.1%)	139 (5.0%)
General events	186 (11.9%)	1 (0.1%)	187 (6.7%)
Event-specific	165 (10.6%)	3 (0.2%)	168 (6.0%)
Object-specific	69 (4.4%)	3 (0.2%)	72 (2.6%)
Lifetime periods	361 (23.1%)	10 (0.8%)	371 (13.3%)
Childhood	97 (6.2%)	0 (0.0%)	97 (3.5%)
Youth	176 (11.3%)	2 (0.2%)	178 (6.4%)
Adulthood	21 (1.3%)	0 (0.0%)	21 (0.8%)
Old age	0 (0.0%)	0 (0.0%)	0 (0.0%)
Semantic content	398 (25.5%)	340 (27.8%)	738 (26.5%)
Artist-related content	386 (24.7%)	314 (25.7%)	700 (25.2%)
General information	23 (1.5%)	43 (3.5%)	66 (2.4%)
Associative detail	412 (26.4%)	98 (8.0%)	510 (18.3%)
Feeling	286 (18.3%)	34 (2.8%)	320 (11.5%)
Association	96 (6.2%)	55 (4.5%)	151 (5.4%)
TV/Radio	66 (4.2%)	22 (1.8%)	88 (3.2%)
Opinion	434 (27.8%)	815 (66.7%)	1,249 (44.9%)
Multiple memories	84 (5.4%)	0 (0.0%)	84 (3.0%)

Only inserts that clearly represented a memory were categorized as MEAMs, and only these inserts were used for further analyses. A total of 157 or 10.1% of MEAM inserts were submitted in the form of audio recordings. MEAM inserts were most commonly assigned to the category of Action memory, and the Action memory sub-categories Singing, Listening, and Dancing. They were next most commonly assigned to Specific details, and the sub-category of Person-specific memories. Of the autobiographical-knowledge categories, MEAM inserts were most commonly assigned to Lifetime periods, then General events and Event-specific knowledge. Within the Lifetime periods category, MEAM inserts were most commonly assigned to Youth, then Childhood, and Adulthood. See Supplemental Material 3 for examples of MEAM inserts assigned to all sub-categories.

Participants could record their memories in writing, as we have seen, or by making an audio recording. Pearson et al. (2023) found that participants are more likely to provide more, and longer autobiographical memories if they can speak them rather than write them. We therefore compared the relative proportions of MEAM inserts provided in spoken and written form that were assigned to each category and sub-category (Table 3) and found that statistically significantly more were spoken (14%) than written (4%) (*p* < .005) for MEAM inserts assigned to Multiple memories. Since a substantial majority of participants had choir-singing backgrounds, we also compared the proportions of MEAM inserts they provided that were assigned to each category and sub-category with those of participants without choir-singing backgrounds but found no differences between the two groups.

### MEAM-insert categories by time of popularity of songs

**Figure 2.**
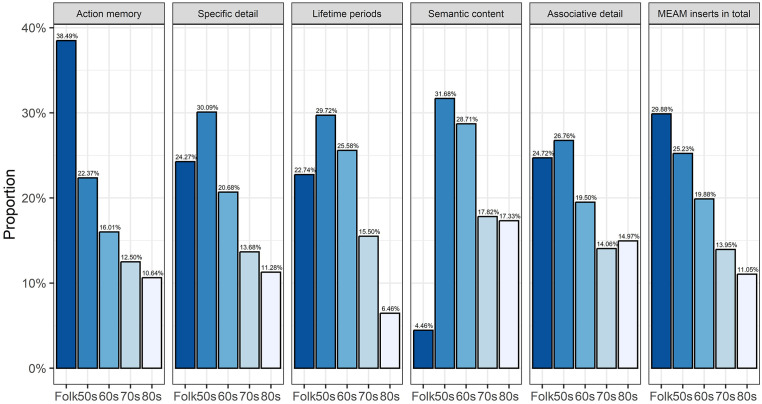
Proportions of MEAM inserts within categories by decades of popularity. *Note.* One insert could be assigned to multiple categories. For folk songs, the number of inserts was weighted by a factor of 1.5. The total numbers of inserts by folk songs and decades of popularity were as follows: Folk songs (after weighting) = 515, 1950s = 434, 1960s = 342, 1970s = 240, 1980s = 190. The total numbers of inserts assigned to the other categories (after weighting of folk songs): Action memory = 912, Specific detail = 586, Lifetime periods = 388, Semantic content = 404, Associative detail = 442. Grand total of MEAM inserts (after weighting) = 1,721.

We compared the proportions of MEAM inserts assigned to each of the five categories representing time of popularity, and folk songs (see Figure 2), the latter having been weighted by a factor of 1.5 as described above. Overall, more MEAM inserts were associated with excerpts from folk songs than from songs popular in any of the four decades. Fewer MEAM inserts were associated with excerpts from songs popular in each successive decade from the 50s to the 80s.

The high proportion of MEAM inserts associated with folk songs, overall, is attributable to the high proportion of MEAM inserts associated with folk songs that was assigned to the category of Action memories. Otherwise, the proportions of MEAM inserts associated with excerpts from songs popular in each of the decades that were assigned to Action memories are similar to the proportions of MEAM inserts associated with songs popular in each of the decades overall. Of MEAM inserts assigned to the other categories, most were associated with songs that were popular in the 50s and fewest with songs that were popular in the 70s and 80s. The proportions of MEAM inserts assigned to the categories of Specific detail, Semantic content and Associative detail associated with songs from these two decades were not very different, but they did differ for the proportions of MEAM inserts assigned to the category of Lifetime periods such that there were much fewer for the most recent decade. Our comparison of the relative proportions of MEAM inserts associated with excerpts from songs popular in each of the four decades and folk songs that had been submitted in writing and via an audio recording yielded no significant difference between the two groups.

### Relationships between the content of MEAMs and emotion ratings

We carried out an exploratory mixed-effects logistic regression analysis to identify the extent to which participants’ emotion ratings for a particular excerpt predicted the content of the MEAM insert associated with that excerpt, operationalized by our assignment of the insert to one or more categories. As shown in Table 4, excerpts rated high for arousal significantly predicted a higher probability of assignment to the sub-category of Youth and a lower probability of assignment to the sub-categories of Singing and Childhood. Excerpts rated high for intensity significantly predicted a higher probability of assignment to the category of Specific detail and the Person-specific sub-category.

**Table 4.** Results of mixed-effects logistic regression analyses.

	Valence		Intensity		Arousal		ID
	Odds ratio (CI 99.5%)	Coefficient (*p* value)	Odds ratio (CI 99.5%)	Coefficient (*p* value)	Odds ratio (CI 99.5%)	Coefficient (*p* value)	Random effect
Action memory	0.94 (0.72–1.22)	−0.06 (.51)	1.02 (0.82–1.27)	0.02 (.80)	0.94 (0.73–1.20)	−0.06 (.47)	0.35
Singing	0.89 (0.66–1.20)	−0.11 (.29)	1.19 (0.93–1.52)	0.17 (.05)	**0.76 (0.58**–**0.99)**^a^	−**0.27 (.004)**^a^	0.31
Listening	1.07 (0.73–1.57)	0.07 (.61)	0.78 (0.57–1.06)	−*0.25 (.02)*	1.24 (0.85–1.80)	0.21 (.11)	0.52
Dancing	0.82 (0.57–1.20)	−0.19 (.15)	1.00 (0.73–1.37)	0.002 (.99)	1.25 (0.88–1.77)	0.22 (.07)	0.59
Performing	1.44 (0.43–4.78)	0.36 (.40)	1.00 (0.43–2.32)	0.003 (.99)	1.19 (0.42–3.38)	0.18 (.64)	2.96
Playing (instrument)	1.88 (0.58–6.03)	0.63 (.13)	0.74 (0.29–1.91)	−0.30 (.38)	0.74 (0.26–2.05)	−0.31 (.40)	5.11
Specific detail	1.05 (0.79–1.39)	0.05 (.63)	**1.31 (1.04**–**1.65)**	**0.27 (.001)**	0.92 (0.71–1.18)	−0.09 (.329)	0.46
Person-specific	1.04 (0.76–1.42)	0.04 (.74)	**1.32 (1.02**–**1.71)**	**0.28 (.002)**	0.83 (0.62–1.10)	−0.19 (.06)	0.34
Location-specific	1.11 (0.70–1.75)	0.10 (.53)	1.14 (0.79–1.64)	0.13 (.32)	1.01 (0.68–1.51)	0.01 (.94)	0.39
General events	0.85 (0.58–1.26)	−0.16 (.25)	1.12 (0.81–1.56)	0.11 (.33)	1.04 (0.73–1.50)	0.04 (.74)	0.77
Event-specific	0.98 (0.64–1.51)	−0.02 (.90)	1.21 (0.85–1.72)	0.19 (.14)	1.03 (0.69–1.53)	0.03 (.84)	0.72
Object-specific	0.81 (0.44–1.50)	−0.21 (.33)	1.04 (0.61–1.75)	0.04 (.85)	1.71 (0.88–3.32)	*0.54 (.02)*	1.07
Lifetime periods	1.15 (0.84–1.58)	0.14 (.21)	1.04 (0.81–1.34)	0.04 (.67)	0.97 (0.73–1.30)	−0.03 (.80)	0.38
Childhood	0.96 (0.60–1.53)	−0.04 (.81)	1.08 (0.73–1.60)	0.08 (.57)	**0.53 (0.34**–**0.82)**	−**0.64 (<** **.0001)**	0.26
Youth	1.27 (0.80–2.00)	0.24 (.15)	0.86 (0.60–1.21)	−0.16 (.21)	**1.58 (1.03**–**2.42)**	**0.46 (.003)**	0.58
Adulthood	0.87 (0.27–2.74)	−0.14 (.73)	1.67 (0.64–4.35)	0.51 (.13)	1.01 (0.35–2.90)	0.01 (.98)	0.83
Semantic content	1.17 (0.84–1.61)	0.15 (.19)	1.05 (0.81–1.37)	0.05 (.61)	1.35 (1.00–1.82)	*0.30 (.006)*	1.03
Artist-related content	1.18 (0.85–1.64)	0.17 (.16)	1.05 (0.81–1.38)	0.05 (.59)	1.31 (0.97–1.78)	*0.27 (.01)*	1.15
General information	1.24 (0.42–3.66)	0.22 (.57)	1.10 (0.48–2.50)	0.10 (.74)	0.92 (0.37–2.27)	−0.08 (.80)	0^b^
Associative detail	0.97 (0.72–1.31)	−0.03 (.77)	1.26 (0.98–1.62)	*0.23 (.009)*	0.87 (0.66–1.16)	−0.14 (.17)	0.53
Feeling	0.83 (0.59–1.17)	−0.19 (.12)	**1.75 (1.30**–**2.36)**	**0.56 (<** **.0001)**	0.88 (0.64–1.22)	−0.13 (.27)	0.85
Association	1.14 (0.67–1.93)	0.13 (.50)	0.78 (0.51–1.20)	−0.24 (.11)	1.04 (0.63–1.72)	0.04 (.83)	0.49
TV/ Radio	1.20 (0.63–2.30)	0.18 (.42)	0.61 (0.37–1.02)	−*0.49 (.007)*	1.20 (0.64–2.24)	0.18 (.43)	0.64

CI = confidence interval.

Significant associations (*p* < .005) are emboldened. Associations with coefficients *p* < .05 are italicized for interest.

a Rounded down to point out the statistical significance.

b Singular fit due to small number of memory inserts assigned to the category.

**Figure 3.**
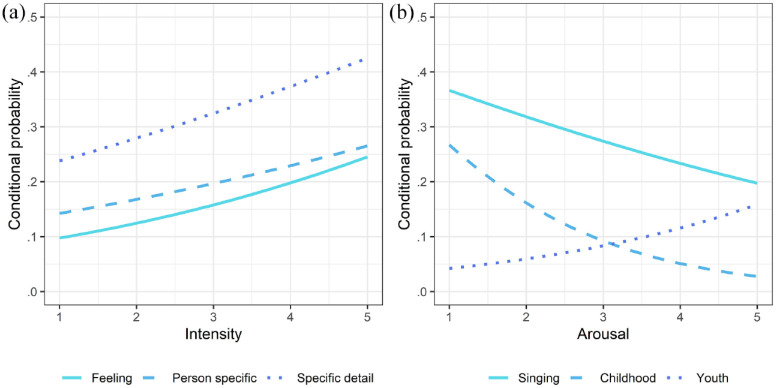
Conditional probabilities of memory-insert categories associated with intensity and arousal. *Note.* The probabilities presented on the y-axis are conditional on the participant having submitted an insert for a particular song. Only statistically significant relationships are shown here. (a) Conditional probability of Feeling (solid line), Specific-detail (dotted line), or Person-specific memory (dashed line). (b) Conditional probability of memory related to Singing (solid line), Childhood (dashed line), or Youth (dotted line).

Statistically significant relationships between the emotion ratings and the conditional probability of the MEAM insert being assigned to a particular category or sub-category are illustrated in Figures 3(a) (intensity) and (b) (arousal).

## Discussion

The aim of the content analysis was to find out what kinds of information and levels of detail are contained in the MEAMs of healthy older adults. We found in our sample that they often involved music-related activity, such as singing, dancing, or listening to music, and the details of specific people or locations. Memories from youth were more common than other life periods, and they were not generally time-specific. Also, the analyses exploring the relationships between emotion ratings and MEAM categories and sub-categories suggest that different kinds of MEAM content have different emotional profiles.

The case-by-case analysis revealed that just over half of the submitted memory descriptions involved active participation in a music-related activity. The most common of these activities was singing, followed by listening to music, dancing, and lastly playing an instrument. Singing and dancing were also commonly reported activities in the MEAMs of college-aged participants (Janata et al., 2007), besides driving, but not listening to music; this may reflect generational differences in the way that music is consumed. It is plausible to argue that listening to music used to be considered an activity in itself, but nowadays when music is so widely available, for example through streaming services and social media content, its consumption has become more routine and passive in nature.

More than a third of MEAM descriptions in the present study included details of specific people, locations, events, or objects. Unlike Janata et al. (2007), who found larger proportions of specific memories in each of these categories, we did not name them. Instead, we gathered free descriptions of evoked memories, aiming to capture contextual information and gain a more comprehensive understanding of the data. While Janata et al. (2007) used Linguistic Inquiry and Word Count software (LIWC2001; Pennebaker et al., 2003) to analyze open-ended responses in each of their categories, we carried out our content analysis manually. This approach may increase the risk of error and subjective rater bias, but it also provides a more comprehensive and ecologically valid way of mapping the content of MEAMs.

We also assigned memories to three categories representing different levels of autobiographical knowledge (Conway & Pleydell-Pearce, 2000) and temporal specificity. Notably, there were more memories referring to lifetime periods than temporally specific events (similarly, Janata et al., 2007, found that their participants recalled twice as many memories of lifetime periods than specific events). This may be attributable to the positivity effect, since it has previously been shown that MEAMs tend to be more positively than negatively valenced (Janata et al., 2007; Salakka et al., 2021). The positivity effect has been found in studies of older adults: focusing on an emotional state when carrying out an autobiographical task produces more positive memories, while focusing on accuracy of recall produces more negative memories (Kennedy et al., 2004). In the context of MEAMs, the emotions evoked by the song may automatically guide the listener to recall positive memories related to lifetime periods as opposed to specific events.

Reports of details as well as time-specific events were relatively frequent in the MEAM data. MEAMs are typically vivid (Belfi et al., 2016, 2022; Jakubowski & Ghosh, 2021), especially when they are detailed. Emotions can also play a key role in the encoding and recollection of details (Kensinger, 2009), and the personal significance of music is strongly associated with emotional intensity (Salakka et al., 2021). In line with this, the results of our mixed-effects logistic regression analyses showed that excerpts rated high for intensity significantly predicted a higher probability of assignment to the category of Specific detail and the Person-specific sub-category. In other words, music associated with (typically positive) emotions can evoke detailed if not time-specific memories. The association between emotion and memory also underlays our focus in the present study on emotions evoked by music rather than MEAMs, as music-evoked emotions include not only the emotions evoked by the music but also the emotions evoked by the MEAMs; they both form individuals’ subjective emotional experiences of listening to music.

Confirming our prediction and adding to the evidence for the reminiscence bump (Jakubowski et al., 2020; Platz et al., 2015; Schulkind et al., 1999; Schulkind & Woldorf, 2005), youth was the most commonly mentioned age-related lifetime period for MEAMs, followed by childhood. Only a few memories were linked to adulthood and none to old age, perhaps because we did not include any songs released since 1990, even though many are still quite popular. Memories of youth were positively associated with arousal while childhood memories were negatively associated with arousal, perhaps because songs from different lifetime periods have different musical content (e.g., tempo) and/or emotional content. Memories related to singing were also negatively associated with arousal. Participants may have experienced less lower arousal when recalling songs from childhood because such songs are typically soothing, sung within the family, and associated with child-caregiver bonding, but this is mere speculation as we only explored associations between childhood memories and singing, respectively, and arousal. Nostalgia—often for childhood—is also typically characterized as a low-arousal emotion (Trost et al., 2012; Van Tilburg et al., 2018), while songs heard in youth are likely to evoke more highly arousing emotions, since they are associated with adolescence and the development of individual and social identity.

Considering that memories from youth and childhood are more frequent than memories from adulthood or old age, a similar age-related pattern would be expected to emerge from the stimulus perspective. We observed a clear trend such that older songs generally evoked more memories than newer songs, although the majority of memories were evoked by folk songs, which were not categorized by decade. Furthermore, a large proportion of action memories was related to folk songs, perhaps because they are commonly sung at school in Finland. Otherwise, most memories were evoked by songs dating from the 50s, and then the 60s, when most of the participants were in their childhood and adolescence. While songs from the 70s and the 80s evoked the fewest memories they still evoked more semantic content than folk songs, which evoked the least amount of semantic content. This may be because they have few semantic associations (e.g., composer or artist) and in many cases their origins are unknown. In short, folk songs and popular (e.g., jazz, rock) songs evoke qualitatively different MEAMs, the former containing more action-related content and very little semantic content.

The findings of the content analyses further revealed that just under a fifth of the submitted MEAMs mentioned a particular feeling or emotion related to an autobiographical memory, which was also associated with a higher rating of the emotional intensity of the song. Again, this supports the close connection between emotions and memories, and evidence for the key role of emotions in the retrieval and encoding of personally salient events (Holland & Kensinger, 2010). Our previous quantitative research found a strong positive relationship between emotional intensity and elicited MEAMs (Salakka et al., 2021) so it is not surprising that in the present study a large proportion of memories included descriptions of associated emotions. Also, a quarter of submitted MEAMs included semantic information about the artist, which is fewer than the MEAMs including action memories. Even so, semantic information relating to the artist could be an important factor when autobiographical memories are evoked by music.

The present study had some methodological issues that warrant discussion. First, participants could submit memory inserts either by making an audio recording or typing in a text box. As found by Pearson et al. (2023), it seems to have been easier to use the first method, as the audio-recorded inserts contained more memories than the typed inserts. Second, participants could submit as many inserts as they liked, and the inserts were not assigned to mutually exclusive categories (as shown in Table 3 and Figure 2). The analyses were therefore biased toward the participants who submitted the most MEAMs. Also, some participants may have misunderstood the instruction “Submit a memory” in the OMRT instructions, thinking that this was compulsory rather than voluntary. However, the 82 participants who submitted inserts did not differ demographically from whole sample of 113, the distribution of MEAM inserts submitted in response to different songs was approximately normal, and the distribution of MEAM inserts submitted by a single participant was close to uniform. Third, the OMRT was a self-report measure. This allowed participants to describe their memories freely, increasing the ecological validity of the study, but the style of the submitted MEAMS varied widely, so the data were heterogeneous. Fourth, we took a data-driven classification approach, limiting the comparability of our results to those of other studies using fixed categories and/or scoring systems for classifying autobiographical memories (e.g., Levine et al., 2002). This was justified, however, because MEAMs differ qualitatively from autobiographical memories, generally (e.g., they include musical actions such as singing), and because there is currently no established and widely used system for classifying MEAMs that takes this into account. Fifth, the majority of our participants were senior choir singers, which may have skewed the results to over-represent the frequency of MEAMs and musical activities reported in them, and potentially limits the generalizability of the findings to the population of older people. Finally, given the idiosyncratic nature of musical preferences, MEAMs would have been elicited ideally by music freely selected by participants because of its personal importance to them. Nevertheless, Kristen-Antonow (2019) found that using stimuli from a researcher-selected pool of music from the national charts effectively elicited younger adults’ autobiographical memories, and we found this approach similarly effective with older adults in the present study. It allowed for greater generalizability than participant-selected music and we did not have to ask participants to tell us about their preferred songs in advance.

In conclusion, our analysis of the content of the MEAMs of the older adults who took part in the study suggests that they often involve some kind of music-related activity such as singing, dancing, and listening to music, and specific details most commonly of people or locations. Music may be particularly effective in evoking general MEAMS linked to lifetime periods (childhood, youth, or adulthood) rather than time-specific events. A relatively large proportion of MEAM inserts included semantic content, which suggests that artist-related information is often evoked by music. The majority of participants’ MEAMS were from youth, illustrating the reminiscence bump in a qualitative context. Finally, participants found the songs they heard more or less arousing depending on the lifetime period with which they were associated. The results of this study go some way toward explaining the unique properties of MEAMs and could be used, for example, to improve and optimize music-based rehabilitation. It would be worth conducting similar, longitudinal and cross-sectional experiments in future with the participation of people with memory disorders to find out if the nature of MEAMs changes over the course of neurodegeneration.

## Supplemental Material

sj-docx-1-msx-10.1177_10298649241297931 – Supplemental material for Exploring the nature of music-evoked autobiographical memories in healthy aging: A mixed-methods study Supplemental material, sj-docx-1-msx-10.1177_10298649241297931 for Exploring the nature of music-evoked autobiographical memories in healthy aging: A mixed-methods study by Mia O’Shea, Ilja Salakka, Anni Pitkäniemi, Emmi Pentikäinen, Petri Toiviainen and Teppo Särkämö in Musicae Scientiae

sj-docx-2-msx-10.1177_10298649241297931 – Supplemental material for Exploring the nature of music-evoked autobiographical memories in healthy aging: A mixed-methods study Supplemental material, sj-docx-2-msx-10.1177_10298649241297931 for Exploring the nature of music-evoked autobiographical memories in healthy aging: A mixed-methods study by Mia O’Shea, Ilja Salakka, Anni Pitkäniemi, Emmi Pentikäinen, Petri Toiviainen and Teppo Särkämö in Musicae Scientiae

sj-docx-3-msx-10.1177_10298649241297931 – Supplemental material for Exploring the nature of music-evoked autobiographical memories in healthy aging: A mixed-methods study Supplemental material, sj-docx-3-msx-10.1177_10298649241297931 for Exploring the nature of music-evoked autobiographical memories in healthy aging: A mixed-methods study by Mia O’Shea, Ilja Salakka, Anni Pitkäniemi, Emmi Pentikäinen, Petri Toiviainen and Teppo Särkämö in Musicae Scientiae
