# Supplementary material for: Exploring the nature of music-evoked autobiographical memories in healthy aging: A mixed-methods study
Source: Music Sci. 2025 Aug 25;29(3):484–501. doi: 10.1177/10298649241297931 (PMC12377674; doi:10.1177/10298649241297931)
Supplement: sj-docx-2-msx-10.1177_10298649241297931 – Supplemental material for Exploring the nature of music-evoked autobiographical memories in healthy aging: A mixed-methods study [file sj-docx-2-msx-10.1177_10298649241297931.docx]

## Supplemental material 2

The old-time music rating task (OMRT) ratings concerning emotional and memory domains translated to English. All domains were rated on a 5-point Likert scale and the range is presented here.

1. Valence (How pleasant did you find the song? Rating: very unpleasant—very pleasant)
2. Emotional intensity (How strong emotions did the song evoke? Rating: no emotions at all—very strong emotions)
3. Arousal (How did the song affect your arousal state? Rating: decreased arousal significantly—raised arousal significantly)
4. Familiarity (How familiar was the song to you? Rating: not familiar at all—very familiar)
5. Autobiographical salience (How much personal memories did the song evoke? Rating: no personal memories at all—significant amount of personal memories)
