# Supplementary material for: Exploring the nature of music-evoked autobiographical memories in healthy aging: A mixed-methods study
Source: Music Sci. 2025 Aug 25;29(3):484–501. doi: 10.1177/10298649241297931 (PMC12377674; doi:10.1177/10298649241297931)
Supplement: sj-docx-3-msx-10.1177_10298649241297931 – Supplemental material for Exploring the nature of music-evoked autobiographical memories in healthy aging: A mixed-methods study [file sj-docx-3-msx-10.1177_10298649241297931.docx]

## Supplemental material 3. Example MEAM descriptions.

| Category/Sub-category | Example descriptions (MEAM) |
| --- | --- |
| Action memory |  |
| Singing | This was always sung in the school choir at the spring festival. The song is associated with the feeling that summer holiday freedom is about to arrive. |
| Listening | We listened to this with our friends from the jukebox in a bar while waiting for the school bus to leave. |
| Dancing | This has been danced too, nice rhythm and Danny, timeless! |
| Performing | I have performed this myself. |
| Playing (instrument) | One of the first songs I learned to play on the guitar in the mid-60s. |
| Specific detail |  |
| Person-specific | My sister and I used to play this album almost every night back then. |
| Location-specific | Reminds me of the trip to Paris I took as an adult. |
| General events | My favorite songs from 70s student discos. |
| Event-specific | We sang this on a group trip in Estonia. |
| Object-specific | We went to see Elvis movies, and his picture was on my wall too. Youth and dreams. |
| Lifetime periods |  |
| Childhood | This reminds me of childhood and mother with her songs. |
| Youth | As a teenager, I was constantly listening to the music of the Beatles, and this song was one of my favorites. |
| Adulthood | Brings a lot of emotions, from the times when I was working in Sweden. |
| Semantic content |  |
| Artist-related content | Tamara Lund has a great voice and I have seen her on the opera stage in the 60s. |
| General information | This song was also a hit when I was little. |
| Associative detail |  |
| Feeling | I sang this as a child, and even when I was a little older, wistfully and passionately in the mood. |
| Association | When listening or singing this, nature trips and summer come to mind and the hope to go on a hiking trip again. |
| TV/Radio | I remember when there was a TV show about Vera Telenius and she performed this on it. |
| Opinion | I remember a lot of occasions where this was playing in the background, a pleasant song, really nice to listen to. |
